# Traditional herbal medicine regulatory implementation in Ethiopia: a qualitative study

**DOI:** 10.3389/fphar.2024.1392330

**Published:** 2024-04-12

**Authors:** Rashed Edris Usure, Dereje Kebebe, Yesuneh Tefera Mekasha, Gemmechu Hasen, Nuredin Chura Waritu, Sileshi Dubale, Sultan Suleman

**Affiliations:** ^1^ School of Pharmacy, Department of Pharmaceutical Chemistry, Hawassa University, Hawassa, Ethiopia; ^2^ School of Pharmacy, Pharmaceutics, Jimma University, Jimma, Ethiopia; ^3^ Department of Veterinary Pharmacy, Pharmaceutical Quality Assurance and Regulatory Affairs, University of Gondar, Gondar, Ethiopia; ^4^ Jimma University Laboratory of Drug Quality (JuLaDQ) and School of Pharmacy, Institute of Health, Jimma University, Jimma, Ethiopia; ^5^ Department of Biomedical Sciences, School of Medicine, Wolaita Sodo University, Wolaita Sodo, Ethiopia; ^6^ School of Pharmacy, Mattu University, Mattu, Ethiopia

**Keywords:** qualitative study, traditional herbal medicine, regulatory framework, implementation, Ethiopia

## Abstract

**Background:** Approximately 80% of the Ethiopian population predominantly depends on herbal medicines (HMs) for their primary healthcare needs. Nevertheless, worries regarding the safety, efficacy, and standard of herbal-based treatments have been escalating due to the lack of strong regulatory frameworks. Therefore, the study aimed to assess the presence of regulatory frameworks for traditional herbal medicines and their enforcement in Ethiopia.

**Methods:** The qualitative–phenomenological study design was conducted from November 2021 to March 2022 G.C. The study included 25 regulatory official key informants (KIs) who work for national and regional medicine regulatory agencies, and 15 traditional herbal medicine (THM) practitioners who work at the regional level were purposefully selected for an in-depth interview (IDI). An in-depth interview guide was developed through the purposive sampling technique. The collected data were analyzed using thematic content analysis techniques.

**Results:** The study found that the current national medicine proclamation is deemed inadequate in the regulation of THM. Both conventional and traditional herbal medicines are regulated by a single agency. Weak legal enforcement, a lack of government commitment and support, resource constraints, and inadequate regulatory tools are the main challenges faced in THM regulation.

**Conclusion:** Overall, the study found inadequate legal frameworks and weak THM regulatory implementations in Ethiopia. Consequently, it is critical for all regulatory authorities in Ethiopia to exert their utmost efforts to effectively regulate THM.

## 1 Introduction

The utilization of herbal remedies and nutraceuticals is experiencing rapid growth worldwide as an increasing number of individuals turn to these products to address diverse health issues within various national healthcare systems (Geneva, 2004). According to the World Health Organization (WHO, 2013), approximately 80% of the population in developing countries uses herbal medicine to meet their primary healthcare requirements. According to a report by Abebe (1986), over 80% of the population in Ethiopia also depends on traditional medicine, and in 2015, more than 95% of the preparations were made from plant origin (Kefalew et al., 2015). The aforementioned statement pertains to the larger portion of the rural population as well as certain segments of the urban population who have limited or no availability of modern healthcare facilities (Wassie et al., 2015).

Although herbal medicines (HMs) play a crucial role in therapeutic settings, there has been uncertainty regarding the safety, effectiveness, and quality of herbal-based products due to the absence of robust regulatory frameworks for herbal remedies, in contrast to modern pharmaceuticals (Ali and Omer, 2012; Suleman et al., 2016). Therefore, the development of a system that enhances the accessibility of traditional herbal medicines with established standards of quality, safety, and effectiveness plays a significant role in ensuring widespread access to high-quality healthcare across the globe (Ekor, 2014). Despite the efforts made by numerous countries to implement an integrated healthcare approach, as outlined in the Alma-Ata Declaration, the WHO report of 2013 reveals that the outcome has been far from satisfactory. These countries have undertaken extensive activities to ensure the provision of accessible, reliable, safe, good quality, and reasonably priced traditional medicine services and products, yet the results have been disappointing (Zhang et al., 2015). Attention toward THM has been on the rise lately, owing to the increasing demand for HM, the depletion of HM sources, and the diminishing traditional HM knowledge and economic value (Goitom et al., 2022). Hence, this creates worldwide concern for traditional/traditional herbal medicines’ policy, regulatory framework, safety, quality, efficacy, access, and rational use.

According to a worldwide survey carried out by the World Health Organization (WHO), approximately 124 member states (64%) indicated that they possessed laws or regulations pertaining to herbal medicines. Conversely, nearly 65% of member states, which amounts to approximately 125 countries, reported having a registration system in place for herbal medicines. In Africa, a significant abundance of traditional herbal medicines exists, accompanied by a wealth of public knowledge regarding their usage. However, the absence of a legal framework prevents the incorporation of these traditional herbal medicines into the existing drug legislation (Sharad et al., 2011). Throughout various regions of Africa, herbal remedies are readily available for purchase in open markets, shops, and even through traditional healers, without the requirement of providing scientific evidence to support their safety, effectiveness, or quality (Sharad et al., 2011; Demeke et al., 2022).

Numerous gaps in policy design and practice have been identified in sub-Saharan Africa, despite the fact that most studies have originated from a limited number of countries, such as Nigeria, South Africa, Ghana, and Uganda (Sharad et al., 2011). In contrast to other nations, Ethiopia lacks comprehensive data on registered traditional practitioners or the registration process within the Federal Ministry of Health. However, herbal medicines are readily available on the streets, with accompanying medical assertions (James et al., 2018). There also exists a lack of clarity regarding regulatory requirements for the manufacturing or safety assessment of traditional and herbal medicines, which are not included in the list of essential medicines. Additionally, there is neither a post-market surveillance system, a restriction on the sale of herbal medicines, nor a guideline for clinical trials using traditional medicines (Teshome, 2017; James et al., 2018).

Ethiopia has granted approval to Proclamation 661 of 2009, which has been subsequently modified by the Ethiopia Food and Drug Administration (EFDA) Proclamation 1112 of 2019, to serve as an authorized framework for the official regulation of traditional herbal medicines (TMs) (Abay, 2009). Nevertheless, the level of practical implementation of these legislations remains uncertain. The implementation of any regulations pertaining to medicines can be influenced by a range of factors, including legal aspects and developmental stage, enhancement of the healthcare system, availability of trained personnel, scale and structure of the regulatory authority, financial resources, political priorities, and other interconnected elements (Ndomondo-Sigonda et al., 2021). As a result, the gaps in THM regulation’s legal basis and the weakness of its execution are identified by evaluating the availability and functionality of THM regulatory activities, outcomes, and tools.

Until now, there has been no comprehensive regional-level investigation into the legal structure and practical execution of traditional herbal medicine (THM) regulation in Ethiopia. A solitary study conducted by Demeke et al. (2022) in Addis Ababa evaluated the policy governing herbal medicine regulation and its implementation in Ethiopia, specifically focusing on the registration process of herbal medicines. According to this finding, Ethiopia lacks specific legislation for protecting indigenous medicinal knowledge, leading to mistrust, obscuring secrets, and an insufficient budget for HMs, resulting in a lack of expertise, education, and modern laboratories. Funders and donors also lack support for the pharmaceutical sector (Demeke et al., 2022). The assessment of challenges faced by medicine regulatory offices and THM practitioners in implementing THM regulations in Ethiopia is crucial in the development of a comprehensive herbal medicine regulatory framework. The effective implementation of this framework plays a crucial role in enhancing the utilization of herbal medicines (Ekor, 2014). Therefore, the objective of this study was to evaluate the current regulatory structure and execution, as well as address the obstacles faced by medicine and health regulatory agencies and THM practitioners in regulating THM products, practices, and practitioners within the traditional herbal medicine environment.

## 2 Materials and methods

### 2.1 Study setting

The study was conducted in the selected Ethiopian region and the capital city of Addis Ababa from November 2021 to March 2022 G.C. Ethiopia is located at the horn of Africa, one of the oldest civilizations and the second-most populous country in Africa with a different range of cultures and languages. Ethiopia shares a border with Sudan and South Sudan on the west, Kenya on the south, Somalia and Djibouti on the east, and Eritrea on the north. The population growth in Ethiopia is characterized by rapid population growth, which is estimated at a 2.6% rate per year. Ethiopia is characterized by a wide range of ecological, edaphic, and climatic factors and a diversity of flora composition. The flora of Ethiopia is estimated to include 6,500–7,000 species, with 12%–19% of medicinal plants (MPs) being endemic to the country (Hiranmai Yaadav, 2013). Traditional medicines play a critical role in Ethiopia, with approximately 80% of the human population and 90% of livestock relying on them for primary healthcare (Hiranmai Yaadav, 2013). Ethiopia has witnessed the remarkable medicinal properties of various plants, which have proven to be highly effective in treating certain ailments in both humans and livestock.

### 2.2 Study design and sampling technique

A qualitative phenomenological study design was conducted from November 2021 to March 2022 G.C. Ethiopia has a federal government administration system that is divided into 11 regional states and 2 city administrations with varied agro-ecological zones. Accordingly, the random sampling technique was used to select the regional states and two city administrations that comprised this study. As a result, two regional states (Oromia and Southern Nations, Nationalities, and Peoples’ Region (SNNPR)) and Addis Ababa City were randomly selected. The federal-, regional-, city-, zonal-, sub-city-, town-, and woreda-level medicine and health regulatory authorities, directorates, and offices were included in the study from Addis Ababa City, Oromia, and SNNPR regional states. In addition, THM practitioners (THMPs) who are practicing in Oromia and Addis Ababa City were included in this study.

The study setting of the THMPs was selected by considering the registration status, number of THM practitioners, and duration of THMPs in the selected study area. Based on this, THM practitioners who practiced in Oromia and Addis Ababa were involved in the present study and were selected from Oromia, SNNPR (South Nation Nationalities and Peoples’ Region), and Addis Ababa.

Consequently, 25 regulatory official key informants (KIs) and 15 THM practitioners were purposefully selected for an in-depth interview (IDI). The regulatory officials consisted of the EFDA, Pharmaceutical and Medical Equipment Directorate (PMEAD), Veterinary Drug and Animal Feed Administration and Control Authority (VDAFACA), Ethiopia Health Institute (EPHI), and Traditional Medicine Healer (TMH) associations. In addition, the regional-, city-, zonal-, sub-city, town-, and woreda-level medicine and health regulatory offices participated as key informants. Similarly, registered THM practitioners were selected from the Oromia regional state and Addis Ababa City.

The study aimed to address its objectives by categorizing the questions into 12 distinct themes. Themes one to eight focused on the overall implementation of herbal medicines, specifically involving regulatory personnel at the national and regional levels. On the other hand, themes one to four delved into the practices of traditional herbal medicine practitioners at the regional level. To delve deeper into the status of herbal policy, regulation, and challenges, the themes were further divided into sub-themes based on their content similarity.

### 2.3 Source of population

The source population consisted of all federal- and regional-level authorities, directorates, offices, case teams, and TM healer associations that were involved in TM and HM regulations. Moreover, the source population for THM practitioners included all THM healers who were registered by the Oromia regional state and Addis Ababa city medicine and health regulatory offices.

### 2.4 Study population

All regulatory officials who were directly delegated to federal- and regional-level authorities, directorates, offices, case teams, and THM healer associations on THM regulatory activities in those selected study areas were included. Additionally, all traditional medicine healers who registered and practiced as THM practitioners in selected zones and towns of the Oromia region and selected woredas of Addis Ababa city were included.

### 2.5 Inclusion and exclusion criteria

#### 2.5.1 Inclusion criteria

The inclusion criteria encompassed regulatory experts who were employed for a minimum of 2 years in the selected offices, case teams, and associations in positions and roles relevant to THM regulation, registration, and inspection. Additionally, THM practitioners who registered and practiced for a minimum of 2 years in selected towns of the Oromia region and woredas of Addis Ababa city were included. The KIs’ experience, delegation, role, and activities on THM regulation in the selected offices, case teams, and associations were intensely considered in order to select regulatory experts.

#### 2.5.2 Exclusion criteria

The study excluded individuals who were not appointed as THM regulatory activity case team leaders in the selected offices and were temporarily delegated as case team leaders during the data collection period, THM practitioners who were subjected to sanctions at the time of data collection, and regulatory officials who were indirectly involved in THM regulation-related activities.

### 2.6 Sample size determination and sampling procedure

In the study, 25 medicine and health regulatory experts from different federal and regional regulatory offices participated. In addition, 15 THMPs who registered and practiced for at least 2 years under selected regulatory offices were involved. The maximum number of study participants was determined by the saturation of information that happened during data collection from the perspective of the study objectives. Regulatory experts were selected purposefully by considering the maximum variation of the participants and their medicines and health regulatory offices.

### 2.7 Study variables

#### 2.7.1 Dependent variables

The policy, legislation, and regulation; system and structure for THM regulatory activities; and THM regulatory implementation outcomes were included as dependent variables.

#### 2.7.2 Independent variables

The functionality of basic THM regulatory activities; human and financial resources; level of regulatory authorities; the existence of regulatory tools (guidelines, standards, checklists, and formats); training and commitment of stakeholders; plan, monitoring, and evaluation system; government and public support; and awareness and belief of regulatory personnel and THM practitioners on THM regulations were included as independent variables.

### 2.8 Data collection methods and procedure

The data were collected through an IDI with purposively selected regulatory officials from selected MHR directorates, offices, and case teams by utilizing unstructured questionnaires using data collection tools adopted from previously published literature records (Goh, 2012; Awodele et al., 2014; Alostad et al., 2019; Chebii et al., 2020; Demeke et al., 2022) and prepared in English with needed modifications.

Similarly, the IDI was carried out with registered THM practitioners selected from the Oromia regional state and Addis Ababa city. Both IDI questionnaires were prepared in the English language with needed modifications. Then, each questionnaire was critically reviewed and edited based on the context of THM regulation’s legal basis and practical THM regulatory activities. Furthermore, language experts translated the THM practitioners’ IDI questionnaires into Afan, Oromo, and Amharic languages. Again, those questionnaires were translated back to English by other language experts in order to check their consistency. Each IDI dataset was collected using unstructured questionnaires to get details and additional, new, and clear information on the present study from each KI, which enabled a strong conclusion. During the data collection, the interviewer recorded audio for volunteer interviewees and took notes for in-volunteer interviewees. Each in-depth interview was carried out with maximum effort, and it took an average of 40 min.

### 2.9 Validity and reliability of the instruments

The validity of data collection tools was proven by availing of both tools that were developed by the principal investigators (PIs) with the comment of four experts: those selected from the EFDA (two experts) and regional regulatory officials (two experts). This is done to ensure that the prepared questionnaires are adequately consistent and representative of the set of questions that enable the study to answer its objectives. In addition, questionnaires for the THM practitioners were translated into local languages (Afan, Oromo, and Amharic) so that they could better understand the message and provide their responses. The translated questionnaire was also provided to Afan, Oromo, and Amharic language medicine regulatory experts to incorporate their comments for clarity and ensure its related meaning with regard to the English version. The PI made the necessary corrections after seeking assistance and recommendations obtained from each regulatory and language expert.

### 2.10 Data quality assurance

The quality of the collected data was assured by utilizing properly designed, commented, corrected, and pre-tested questionnaires for study data collection. In addition, the seated dimensions and elements for both in-depth interviews and critically reviewed questionnaires were able to assure the quality of the collected data. Moreover, unstructured questionnaires were used to collect IDI data, which helped ensure the quality of the collected data since the interviewers were able to further clarify the responses provided by interviewees. In addition, the volunteer participants’ audios were recorded, and the responses of in-volunteer participants were taken in a notebook. Furthermore, the quality of all collected data was assured by using experienced data collectors, conducting close supervision, providing data collectors with field guides, reviewing and checking collected data every day, and using audiotape recorders and notebooks.

### 2.11 Data analysis and presentation

The qualitative data collected by IDI were analyzed using a thematic content analysis approach. All data obtained by handwritten field notes and audio recordings were transcribed into Microsoft Word version 2010. Subsequently, the transcribed data were organized, coded, and compiled into themes and sub-themes based on the similarities of ideas in the responses. Then, the coded themes and sub-themes were sorted, and according to their respective codes, the obtained data were summarized, analyzed, and presented in thematic content analysis techniques within the context of their corresponding themes and sub-themes.

## 3 Results

### 3.1 Qualitative finding from medicine regulatory personnel

The in-depth interviews were conducted with 25 KIs selected from agencies and directorates of Ethiopia’s MoH, regional health regulatory (RHR) offices, VDAFACA, and THM associations. The study participants were 13 pharmacists, 5 public health officers, 2 nurses, 2 environmental health professionals, 2 herbalists, and 1 veterinary doctor. The study participants’ work experiences ranged from 4 to more than 20 years. Approximately 17 KIs were selected from RHR offices, whereas 6 interviewees and 2 herbalists involved in the study were nominated from federal institutions and TM associations, respectively.

### 3.2 Theme 1. Policy, proclamation, and regulation

The majority of interviewees stated that the contents of the current national medicine proclamation on THM regulation are insufficient and inadequate. The absence of ambiguous THM practice and product definition, vagueness of the proclamation on THM regulatory functions, and lack of a framing organization structure that would be desirable for TM regulation were redundantly stated reasons for the inadequateness of the current proclamation.

Some KIs stated that the current proclamation is adequate if it is supported by different TM directives and regulatory tools. One participant (FA-P8) thought that

*“The WHO recommends exclusive policy and law for THM regulation; however, in the EFDA proclamation, both traditional and conventional medicines (CMs) are recognized holistically as medicines for regulation. However, implementing the same regulatory requirements for both CM and TM regulation is difficult.”*

The majority of RHR participants also expressed concern about the inadequacy of regional TM directives on THM preparation and practice regulation.

One participant (SZ-P12) specified that

*“The regional THM directive stated more about the requirements of premises and practitioners' regulation, while THM services and HMs that are prepared by healers are insufficiently stated.”* In addition, most of the RHR interviewees noted the inconsistency of regional states’ THM directives among each other. Regarding this, one interviewee (FP-P5) said that

“*Due to a lack of uniformities among regional THM directives, the EFDA has drafted a THM model directive to solve the problem (KIFA5).”*

Concerning veterinary THM regulation, one KI stated that

“*Regarding TH regulation, our authority (VDAFACA) still has not performed any tangible work other than considering it for future planning; currently, there is no authorized legal framework on veterinary THM regulation (*FA-P24).”

### 3.3 Theme 2. Administrative structure of THM regulation

According to the interviewees, HM products are regulated by the EFDA, and THM practices and practitioners are regulated by RHR offices. The THM regulations are enforced by CM regulatory offices, and they are not analogous in each regional state. One participant claimed that

“*Due to the complaint and accusation that THM practices and products are difficult to approve by research, regulating THM and CM within one framework is so challenging* (FA-P2)."

The majority of the participants stated that the regulatory offices do not have independent budget allocation, human resources, or facilities for THM regulation. Additionally, key informants mentioned that

“*Currently, the EFDA has proposed the Job Evaluation and Grading (JEG) system to establish an exclusive organization structure for TM product regulation* (FA-P2 and FA-P8).”

RHR interviewees specified that the THM practices, practitioners, and premises regulations were enforced at different levels of RHR offices. “In our region (Oromia), THM practitioners, practices, and premise registration and licensing have been performed since 2007 E.C. As per key informants, “*the registration is conducted at the regional health bureau, while inspections are carried out by zonal-, town-, or woreda-level regulatory officials* (OR-P7).” Furthermore, *all traditional herbal medicine regulation-related activities in Addis Ababa are operated at the woreda level* (SA-P15), as cited by a key informant.” The indicators stated by the majority of KIs for the presence of ineffective THM regulatory administrative structures are the inconsistency of THM regulatory structures and systems in regions; the absence of a system linking federal and RHR offices; and the absence of a solid exclusive TM administrative division at the federal level.

### 3.4 Theme 3. Implementation of THM product regulatory functions

The EFDA is currently engaged in promising activities related to TM product regulation. The participants involved in IDIs from federal institutions provided information about the exclusive policy, proclamation, directives, guidelines, checklists, and other formats that have been drafted by the EFDA for THM product regulation. In addition, most of the RHR interviewees reported that category 1 and 2 THM products were not adequately regulated in their region. Additionally, a key informant stated that

“*A THM product regulated by the EFDA is the product that is processed, extracted, quantified, identified, packaged, labeled, and manufactured for market purpose (category 3 and 4 products)(*FA-P3).*”* Additionally, one KI stated that *"currently, it seems that the regulation of TM products prepared by practitioners does not get enough attention* (AA-P9).*"*

One key informant mentioned that

“The TM product regulatory activities that are currently implemented in Ethiopia are inadequate, not a single THM product has received MA yet, and no manufacturer has been licensed to invest in THM production (FA-P2).”

Some IDI participants also remarked that the current TM product policy, proclamation, and regulation did not consider the awareness, skill, knowledge, interest, and commitments of TM practitioners. However, other respondents believed that the legal benchmarks were appropriate and adequate for TM product regulation if implemented effectively. One participant stated that

“Without identifying the knowledge, capability, and interest of our THM practitioners, the provision of GMP training for THM practitioners is not acceptable at all (MF-P8).”

### 3.5 Theme 4. Implementation of THM practice and practitioners’ regulatory functions

Interviewees provided information about the registration and licenses of THM practitioners and practices issued at woreda health offices (in Addis Ababa) or regional health bureaus (in other regions). Most of the RHR IDI participants reported that they regulated THM practitioners, THM premises and their sanitation, THM practice, THM marketing, and other issues that are stated in regional THM directives. In addition, the majority of zonal and woredas KIs narrated inspections of THM service-rendering establishments conducted by them. “*Currently, when we inspect the THM establishment, we ask them (THM practitioners) what they treat rather than what products they use to treat their patients* (WA-P23).*"*

Study participants commonly mentioned the presence of quack THM healers, HMs adulterated with CMs, HMs sold on the streets, and few registered THM healers. Currently, THM regulation in Ethiopia is focused on encouraging practitioners to practice THM under legal supervision rather than imposing strict THM regulation, as some KIs stated. Most interviewees described that the registration and regulation of urban THM practices and practitioners are good. However, those KIs implied that they would have a lot of work to do in rural areas across the country. One participant (MF-P3) thought that

“since THM practices and practitioners’ regulation are ignored by RHR bodies of several regions, it is difficult to say confidently that we performed genuine work on THM practice and practitioner regulation to protect the majority of our communities that use THM nationwide.”

### 3.6 Theme 5. Regulatory capacity of regulatory authorities on THM regulation

#### 3.6.1 Subtheme 5.1. Resources and facilities for THM regulation

Key informants noted a shortage of trained human resources, financial sustainability, laboratories, and facilities needed for THM development and regulation. Most of the RHR participants expressed that the training provided on TM regulation is not sufficient. Five RHR offices informed that they had not received training on TM regulations.

One participant said that

“*There is a shortage of finance and human resources as the budget allocated for CMs and regulatory personnel hired on CM regulation is currently utilized for TM regulation in most medicine regulatory offices* (ST-P18)." On the national level, *"both the EFDA and EPHI laboratories are capable of testing many TM products; they are sufficiently organized with automated equipment*, *such as HPLC*, *GC*, *and TLC* (KIP5)." Again, *"all regional states share one or two laboratories that are found in the capital city* (RO-P14).”

### 3.7 Subtheme 5.2. Regulatory tools for THM regulation

Most of the regulatory tools needed for THM product regulations are now in the draft stage, as informed by all KIs who participated from federal institutions. One participant stated that

“*Now, the EFDA actively works on preparing different regulatory tools, directives, trained regulatory personnel, and fulfilling facilities needed for THM product regulation* (MF-P 3).” Regarding the national pharmacopeia and monographs*, "Ethiopia has authorized the use of BP*, *USP*, *China pharmacopeia*, *and other international standards due to the absence of legally binding national pharmacopeia and monographs* (FA-P8)."

One study participant said that

*“Now, five to six plants are on track to get their own monograph. Currently, we (different scholars) have started discussions on preparing the national HM pharmacopeia* (FP-P5).” A regional health regulatory participant reported the inadequateness of current TM regulatory inspection checklists. Those participants stated that the THM checklists vastly addressed the space, quality, and sanitation of premises, the list of diseases that healers want to treat, practitioners’ personal profiles, and the availability of necessary equipment and materials in the establishments. *“To register practitioners, we ask more about their experiences, acceptability by the community, truthfulness, and education status, and the checklist is more concentrated on the sanitation and quality of the premises* (WA-P22).”

### 3.8 Theme 6. Cooperation and collaboration of stakeholders on THM regulation

Interviewees who participated from the MoH, EFDA, and EPHI stated that the institutions established under the MoH are coordinated by many stakeholders. In addition, those KIs notified that they commonly work together on preparing a TM regulatory framework, regulatory tools, strategic plans, monitoring and evaluation, research, training, promoting the knowledge of TM healers, and other issues. A respondent mentioned that

“*All stakeholders have review meetings every three months to review different THM-related activities and issues available for discussion and evaluation* (MF-P 8).”

A key informant mentioned, *“due to resource constraints and overlap of THM-related activities, the MoH is currently challenged to work with all stakeholders (FM-IP3)*.”

Most RHR participants specified that the cooperation and collaboration of RHR offices with other stakeholders are insignificant. However, the majority of RHR officials described that they have relatively good cooperation with the MoH and TM associations. An interviewed study participant mentioned that

“The regional health regulatory offices are still poor regarding working with scientific communities (OZ-P17).”

### 3.9 Theme 7. Government support

Sixty-four percent of those polled said that the government’s commitment and support for TM/THM regulation are positive. According to KIs, the commitment of the government is expressed by including THM in 5-year strategic plans, preparing a draft of an exclusive TM policy and proclamation, different directives, resource allocation, and supporting TM research and regulation. Additionally, the participants narrated that the government showed support by providing training for regulatory personnel and THM healers. One study participant said that

*“The Ministry of Education started delivering THM as a course in some medical schools and higher institutions, as well as preparing a TM learning module for elementary students (FP-P5)*.”

### 3.10 Theme 8. Challenges existing in THM regulation

#### 3.10.1 Subtheme 8.1. Challenges reported by federal institution KIs

A key informant mentioned that “traditional healers are mistrusted by researchers and the scientific community.” Additionally, other factors are the low interest and dedication of TM healers to modify their practices and products, shortage of trained human resources, finance, facilities, and quality control laboratories, and inadequateness of different THM regulatory tools or instruments. One key informant mentioned that

*“There is an absence of a clear job description, monitoring, and evaluation among stakeholders; nationally, there is a lack of well-organized organizational structures for TM regulation. The regulation of THM products that are prepared by healers (1 and 2 categories) is undermined. The EFDA sometimes delays responding to applicants, e.g., clinical trial applicants, weak implementation of the THM regulatory plan that was drafted on paper into action, and the lack of a training plan for regulatory personnel on TM product regulation.” These were challenges reported by federal institutions’ workers in the traditional medicine regulation* (FA-P4).

#### Most key informants cited that

“The knowledge gap between healers and conventional medicine professionals, weakness in implementing the knowledge and experiences obtained from training to work, absence of committed, accountable, and representative TM healers’ associations, and weak commitment, support, and agreement between different TM healers’ associations (OR-P1, WA-F5, and MF-P8) are other factors that contested the traditional medicine regulatory environments.”

### 3.11 Subtheme 8.2. Challenges reported by regional health regulatory KIs

In this qualitative investigation, the measurement of challenges faced by the regional health regulatory level in regulating traditional herbal medicines was conducted. According to key informants who are employees of regional health regulatory bodies, there exist multiple challenges that influence the effectiveness of regional health regulatory workers in enforcing traditional herbal medicine regulations. A key informant mentioned that some traditional herbal medicine practitioners are not interested in registration, mix their THM practice with spiritual healing services, hide their knowledge, and do not print all the information needed on the product label. In addition, the lack of human resources, finance, research institutions, and quality control laboratories were challenges encountered in the regulatory herbal medicine environment.

Furthermore, the absence of appropriate regulatory mechanisms for category 1 and 2 THM products, insufficiency of various THM regulatory tools, such as the existing THM checklists, inadequate training and support provided to healers and RHR workers, fragmented TM regulatory activities and information, and lack of an organizational structure, legal enforcement, and regulatory attention toward THM have a critical impact on the regulation of herbal medicines. Additionally, the absence of collaboration, monitoring, and evaluation, as well as a training plan for THM regulation, lack of knowledge, dedication, and interest among regulatory personnel, insufficient encouragement of registered THM practitioners by the public and government, and limited public awareness and reporting on THM-related irrational activities were all factors that had an impact on the regulation of herbal medicines.

### 3.12 Qualitative findings from traditional herbal medicine practitioners

A total of 15 registered THM practitioners from the Oromia region (n = 9) and Addis Ababa city (n = 6) participated in the study. All of the respondents were male, and the majority of participants were between 51 and 65 years old (46.7%). Nine KIs had less than 4 years of experience when they registered, and only six respondents had writing and reading skills (Table 1). All KIs currently practice in urban areas and do not have fixed payments for the services they provide. Religious preachers and books are the main sources of THM knowledge for most KIs (6; 40%). The majority of clients visited THM facilities after receiving CM treatment.

**TABLE 1:** Background profile of THM practitioners and their practices, Ethiopia, 2022 (n = 15).

Variable		Frequency	Percentage (%)
Age of respondents (years)	35–50	3	20
	51–65	7	46.7
	>65	5	33.3
Experience (years)	10–20	8	53.3
	21–30	5	33.3
	>30	2	13.3
Experience after registration (years)	<4 years	9	60
	≥4 years	6	40
Educational status of respondents	Read and write	6	40
	Secondary school	4	26.7
	Diploma and above	5	33.3
Main source of knowledge of THMPs	Family members	4	26.7
	Religious preachers and books	6	40
	Reputable THM practitioner	4	26.7
	Self-taught through trial and error	1	6.7
Category of customers who visit THM facilities	After receiving allopathic medicine	12	80
	Before receiving allopathic medicine	3	20

## 4 Theme 1. Awareness and belief of THM practitioners on the regulation of THM

Out of a total of 15 interviewees, 10 notified that they roughly knew the laws and regulations that govern TM products and practices. However, most of the KIs informed that they were not aware of the detailed provisions and prohibitions stated in the regional TM directives. Furthermore, they stated that they were not aware of some prohibited practices that might subject them to penalties. Approximately 8/15 practitioners agreed with the appropriateness of current legal benchmarks and the helpfulness of THM regulation. On the other hand, approximately 60% (9/15) of KIs supported the self-regulation of THM, while most KIs wanted the THM regulation case teams to include TM practitioners (dual-regulation). Nine individuals noted that the current THM registration and license requirements are not difficult for healers. However, some of the interviewees believed that the current requirements were difficult for THM healers.

### 4.1 Theme 2. Quality, safety, and efficacy of the HM monitoring mechanism

The majority (73.3%, 11/15) of KIs specified that they usually used different parts of medicinal plants, minerals, and animal body parts. Some of the study participants reported the utilization of different semi-processed synergic products mixed with natural products (HMs). All interviewees confirmed the absence of uniform procedures and methods they utilized to collect, prepare, and dispense their HM products. The study participants collected medicinal plants from their own gardens and rural areas or purchased them from the nearest markets and abroad. One respondent informed that

*“The herbal medicines were collected from a rural area approximately 550 to 650 kilometers away from Addis Ababa* (KI-5).”

Most (80%) of the study participants expressed that medicinal plants are collected and prepared by themselves, while three KIs informed that sometimes they are operated by other people. The majority (9/15) of interviewees reported that they have full information about the environment in which those harvested medicinal plants grow. Aa key informant stated that

*“Despite the availability of information with regard to the environment where herbal medicine is located and the distance of herbal medicines from the community, having all the information was a pitfall challenge* (KI-2).”

The season and time (morning and afternoon) considerations were considered for HM collections by all interviewees. However, some practitioners noted that they also considered a day (Wednesday or Friday), a collector (sanctified person), and prayed on collected medicinal plants. All study participants described the harvested HM as being transported by experienced people with high caution. Depending on the nature and storage condition, most of the HMs expire between 1 month and 1 year, as most of the KIs stated. Study participants informed that they usually checked the expiration of HMs through visual inspection, smell or flavor, appearance change, tests, and other physical aspects of HMs.

The bottles containing the herbal medicines were commonly found on the shelf, according to most practitioners. However, the label lacked specific product information, notably the product name. One key informant mentioned that “*the reason for the lack of labeling of the product information was to protect the products from theft (KI-14).”* The majority of the KIs (12 out of 15) expressed that they were not given an opportunity to engage in research related to HMs. Only three participants informed about their chance to collaborate with the scientific community in conducting research. All the participants acknowledged the existence of HM products currently available in the market, but none of them were registered with the EFDA or verified by an authorized laboratory for their intended quality, safety, and effectiveness.

### 4.2 Theme 3. THM practice regulation

Hemorrhoids, gynecology cases, nerve cases, cancer cases, internal medicine cases, impotence, evil eyes, dermatology cases, and STI cases are ailments that are commonly treated in the facilities of the practitioners. All participants have a patient registration book in their facilities. In the patient registration book, patients’ names, sex, age, card number, dates of visitation, and addresses are usually recorded, whereas aliments, product types, payments, and follow-up appointment schedules are recorded occasionally. Patient diagnosis often takes into account various types of information, including signs and symptoms, as well as the name of the disease provided by the patient, as stated by most key informants. Despite these circumstances, there is a difficulty in diagnosing patients. A particular informant highlighted that when it comes to diagnosing patients, “*we, as healers, typically commence by considering the type of disease and the symptoms that our patients relay to us (self-diagnosis) (KI-13).”*

More than half of the interviewees noted that they provided information for patients on the type of restricted foods and drinks, the dose and frequency of HM products, and other related issues. Approximately 33.3% (5/15) of KIs were informed that they also advise patients to continue the restriction that CM professionals tell them. The interaction of HM products and allopathic medicine concerns only 40% of KIs while they advise patients. Most practitioners claim that they strongly follow their patients’ healing progress through different techniques. Regarding patient referring, the majority (9/15) of KIs said that sometimes after their patents enable them to be treated by THM, they refer to CM healthcare, while six practitioners think that they have not referred any patients yet. Almost all participants noted that the HM was sold in open areas in sunlight, on the street, and in public places without restriction, especially in woreda towns. According to the interviewees’ estimations, approximately 80%–90% of healers found nationwide are still practicing THM officially without being registered and licensed by their respective health regulatory offices.

Most of the time, patients engage in promoting and advertising THM practice, as stated by the majority of key informants. In addition, the interviewees informed that media, banners, brochures, pamphlets, and stickers were used to promote their services. The majority (80%) of study participants said that they mistrusted scientific communities because they feared that those individuals might steal their products. More than half of the practitioners said that they reported the activities performed in their organization within the provided schedule to their respective regulatory offices. However, some (7/25) of the KIs noted that they did not report within the stated schedules to their respective regulatory offices.

Most practitioners reported that regulatory personnel visited their facilities three to four times annually. Some (4/15) of the KIs informed that inspectors visit once a year to check whether they have renewed their working licenses. Almost all informants reflected that they get information from regulatory officials only when they inspect their facilities. The herbalists stated that most of the time the inspectors showed a negative attitude during the inspections of their establishments.

### 4.3 Theme 4. Formal and informal THM activities

In this study, the traditional herbal medicine practices, formal and informal, carried out by licensed practitioners were evaluated. As per the current result, THM products and practices related to formal and informal activities were carried out in both the Oromia region and the city of Addis Ababa. The details of activities carried out by registered traditional herbal medicine practitioners are described in Table 2.

**TABLE 2:** Formal and informal THM activities practiced by registered practitioners (n = 15).

Formal THM activity	Region	Informal THM activity	Region
THM product and practice-related formal activity		THM product- and practice-related informal activities	
Harvested HMs from nearest well-known rural areas, receive fresh HMs from village-based herbalists, and having good awareness about environment HM grow-in	In both regions	Harvested HMs from rural areas that are 350–650 km far, used unprotected, wild, re-harvest plants, and had poor awareness about environment HM grow-in	In both regions
Used natural product, such as medicinal plant parts, minerals, and animal body parts	In both regions	Used products prepared by other healers and processed and semi-processed products	In both regions
All HMs are prepared and packed by licensed THM practitioners in bottles and then labeled and put on the shelf	In both regions	HMs were prepared by other people, packed in fertilizer bags and papers, did not have labels, and were put on tables or all in one drawer	In both regions
Considered time (morning and late-night) and season (plant part fit for medicine) to collect medicinal plants	In both regions	Considered days (Friday and Wednesday), person (sinless), and prayed on plants prior to collection	In both regions
Provided service only for registered diseases, deliver only HM treatment, and reported within the stated schedule	In both regions	Provided service for unregistered diseases, mixed with spiritual healing, and did not report within the stated schedule	In both regions
Provided proper advise on side effects and labeling using simple dosages of prescribed medicine for patients	In both regions	No provision of proper advise on side effects and dispensed medicine for patients only with verbal instructions	In both regions
Asked patients about medication history and advice on possible interactions prior to HM product dispensing	In both regions	Did not ask patents about medication history and poor patient counseling on the restriction of CM, food, and drinks	In both regions
Provided their services, sold medicinal plants in their THM establishments, and used appropriate words on their promotion and advertising	In both regions	Opened THM service branches, changed work place frequently, sold HMs on the street, did not renew license on time, and used improper words on promotion and advertising	In both regions
Used their own dose adjustment unit and materials for each product dispensed after cleaning properly	In both regions	Used CP dose adjustment unit like capsules and bottles without cleaning properly and did not use gloves on product preparation	In both regions
Medical decisions are based on patient history, subjective and objective facts, and other own techniques	Rare in both	Medical decisions are based on their experience and apprenticeship and patients self-diagnosis	In both regions
Delivered their THM services in premises that fulfilled the standard in number and sanitation	Rare in both	Delivered THM services in premises that did not fulfill the standard in number and sanitation	Rare in both
Report to their respective regulatory offices within the schedule (Oromia (monthly) and Addis Ababa (quarterly))	In both regions	Did not report at all or did not report within the stated schedule to their respective regulatory offices	In both regions

### 4.4 Theme 5: Challenges and supports

#### 4.4.1 Subtheme 5.1. Challenges specified by THM practitioners

Participants were asked to describe the challenges related to herbal medicine practices. The participants asserted that traditional herbal medicine products and services did not get the desired recognition, attention, and support. Additionally, distinguished healers do not get appropriate support, respect, and appreciation from responsible bodies. One participant stated that

“Traditional experts have no official role in the regulation of traditional medicine or traditional herbal medicine, and the products and services of traditional medicine are undermined and disparaged by professionals in the field of biomedicine (KI9).”

During the interview, the key informants also inquired about the government’s activities and clinical services’ interest in incorporating traditional medicines into the national healthcare system. One of the participants indicated that

“The weakness of the government in changing TM-related plans and strategies into practice and the absence of clinical or service integration of TM in the national healthcare system are the critical challenges that contest the practice of herbal medicine practitioners (KI1).”

The majority of the participants acknowledged that the income derived from traditional medicine (TM) practice is insufficient and does not align with the demanding nature of the work. Additionally, they expressed concerns about the lack of support from scientific communities regarding patent issues related to herbal medicine, as well as the inadequate regulation of services provided by unqualified practitioners and unregistered healers in the traditional herbal medicine field. Furthermore, the improper harvesting of medicinal plants by the community for various purposes, the limited investment and financial support from both the government and private sector for THM services, the lack of public support for registered THM practitioners, and the shortcomings in THM regulation were all identified as significant challenges that impact the practice of traditional herbal medicine practitioners.

### 4.5 Theme 5.2: Support needed by THM practitioners

In this study, traditional herbal medicine practitioners were inquired about the necessary assistance required to ensure the successful execution of their practice within the herbal medicine setting. The key informants highlighted the importance of various forms of support. These include assistance in the development and promotion of their current practical activities, support from the government through the implementation of plans, strategies, and advocacies of traditional and herbal medicine (THM), provision of training, finance, materials, and equipment for their services, and the establishment of a formal clinical or service integration of traditional medicine in the national healthcare system.

Furthermore, the key informants confirmed that the essential support required includes offering structured and comprehensive training on traditional herbal medicine (THM) practices, ensuring access to suitable garden areas or land for cultivating and preserving medicinal plants, implementing efficient regulations to monitor unqualified THM healers, endorsing the establishment of robust national associations and training institutions for traditional herbal medicine, and enacting a law that prioritizes the protection of patent rights for THM healers and fosters trust between healers and the scientific community.

## 5 Discussion

The lack of specific policies, laws, and regulations for THM could lead to ineffective enforcement of THM regulatory measures due to overlapping functions, powers, and responsibilities with conventional medicines (Ijaz et al., 2016). Nevertheless, the EFDA has recently formulated a distinct policy and declaration specifically for THMs. This endeavor necessitates the involvement of researchers in order to assess the current state of implementation, legal foundation, obstacles, and the necessary assistance required for regulatory personnel and herbal medicine practitioners driving the establishment of an independent herbal medicine regulatory framework.

In this study, in-depth interviews were conducted with 25 key informants selected from agencies and directorates in Ethiopia and 15 key informants selected from traditional herbal medicine practitioners registered in the regions of Oromia and Addis Ababa City. The participation of diverse regulatory and traditional healer professionals in the execution of policies within a regulatory setting significantly influences the prioritization of objectives and the acceptance of a policy that delineates standards for addressing any challenges in establishing a regulatory framework and resolving the matter of implementing herbal medicine regulations (Fokunang et al., 2011).

The actual study revealed that the majority of study participants stated that the contents of the current national medicine proclamation on THM regulation were insufficient and inadequate. The absence of ambiguous THM practices and product definitions, the vagueness of the proclamation on THM regulatory functions, and the lack of a framing organization structure that would be desirable for THM regulation were redundantly stated reasons for the inadequateness of the current proclamation. Some of the KIs stated that the current proclamation is adequate if different THM directives and regulatory tools support it. However, in the EFDA proclamation, both traditional and conventional medicines are recognized holistically as medicines for regulation (EFDA, 2019). However, implementing the same regulatory requirements for both CMs and THMs is difficult. As per the findings, the herbal medicine regulation system deviated from the WHO recommendation that exclusive policies and laws are required for traditional medicine regulation (Cordell, 2012). This revealed that the THM policy and proclamation are still in the draft stage. The lateness of the THM policy and proclamation may be due to the negligence of the EFDA and other stockholders. The first step in medicinal product regulation is the establishment of regulated product categories and corresponding regulation forms for each category (Picking and Vandebroek, 2019).

As per this finding in Ethiopia, the implementation of THM product regulation was weakly performed when compared with China (Thakkar et al., 2020), South Africa, Ghana, and Uganda. As a result, the presence of legislation and regulation is meaningless if basic regulatory functions are not implemented effectively (Scott, 2004). Hence, it is authoritative for the Ethiopian FDA to demonstrate its dedication toward enhancing the implementation of regulatory practices for traditional medicine products. This can be achieved by addressing the challenges associated with organizational structure, resource allocation, facility management, and regulatory frameworks and formats.

Furthermore, a significant number of participants from the RHR offices also articulated their worry regarding the insufficiency of regional TM guidelines pertaining to the preparation and regulation of THM. They highlighted that the regional traditional medicine directive primarily focuses on the regulations for premises and practitioners, neglecting the necessary attention toward THM services and HM prepared by healers. Furthermore, the majority of the RHR interviewees highlighted the lack of consistency in the THM directives issued by different regional states. In response to this issue of non-uniformity, the EFDA has developed a model THM directive aimed at resolving the problem. The regional health regulatory bodies stated that the VDFACA has not carried out any significant responsibility other than evaluating its plans in relation to traditional herbal medicines for veterinary use. Currently, there is no approved legal framework in place for the regulation of these veterinary THMs. The same practice is performed in Uganda, where both human and veterinary herbal medicines are controlled by the National Drug Authority (UNDA, 2021).

An assessment was carried out to evaluate the administrative structure governing traditional herbal medicine. Consequently, the EFDA was assigned the task of regulating herbal medicinal products, whereas regional health regulatory offices were entrusted with the responsibility of overseeing traditional herbal medicine practices and practitioners. Furthermore, the enforcement of TM regulations falls under the jurisdiction of CM regulatory offices, which vary across different regional states. As highlighted by study participants, the integration of THM and CM within a single framework poses significant challenges due to the difficulties in approving THM practices and products through research. Similar studies reported from Kenya (Chebii et al., 2020), Tanzania (Mujinja and Saronga, 2022), and Zimbabwe (Usai et al., 2021) reported the presence of suspicion between THM practitioners and the scientific community, which this study also identified as a challenge for TM regulation. Indeed, in the current investigation, both professionals and researchers held each other accountable for the cause of doubt, a finding that aligns with a similar study conducted in Ethiopia in the past (Fantaw and Kefale, 2020).

Most of the respondents expressed that the regional regulatory agencies lack autonomy in terms of budget allocation, workers, and infrastructure for traditional herbal medicine regulation. Moreover, experts highlighted that the EFDA has recently suggested the implementation of the Job Evaluation and Grading system to create a dedicated organizational framework for TM product regulation. This indicates the necessity for enhanced government backing to boost the regional regulatory offices through budget allocation, human resource training, and the provision of facilities to advance the regulation of traditional herbal medicine. The Ethiopian EFDA and other regulatory bodies ought to acquire knowledge from Ghana’s herbal medicine industry in terms of its potential, obstacles, and future strategies, taking into account the perspective of advancing the herbal medicine regulatory system (Asase, 2023).

Since 2007 E.C., the regulation of THM practices, practitioners, and premise has been implemented by various regional health regulatory bodies. According to key informants, registration is conducted at regional health bureaus, while inspections are carried out by regulatory officials at the zonal, town, or woreda levels. In Addis Ababa, all traditional herbal medicine regulation-related activities are managed at the woreda level. The study participants highlighted that the ineffective THM regulatory administrative structures are primarily caused by the inconsistency of the TM regulatory structure and system across regions, the lack of a system connecting federal and RHR offices, and the absence of a dedicated THM administrative division at the federal level. The lack of regulatory enforcement allows unregistered practitioners to freely sell herbal medicines in public areas on the street, highlighting the inadequacy of regulations governing the sale of these products (Sharad et al., 2011).

The EFDA is currently engaged in promising activities related to TM product regulation. The participants involved in IDIs from federal institutions were informed that exclusive policies, proclamations, directives, guidelines, checklists, and other formats have been drafted by the EFDA for THM product regulation. In addition, most of the regional health regulators reported that category 1 and 2 THM products were not adequately regulated in their region. Additionally, THM products regulated by the EFDA are the products that are processed, extracted, quantified, identified, packaged, labeled, and manufactured for market purposes (category 3 and 4 products). The findings revealed that the current regulatory measures for THM products in Ethiopia are insufficient. Despite this, there has not been a single THM product that has obtained market authorization, and no manufacturer has been granted a license to invest in the production of THM products. A comparable situation was observed in Tanzania, where both market authorization and licensed manufacturers were lacking (Mssusa et al., 2023).

The current traditional herbal medicine product policy, proclamation, and regulation were criticized by study participants for not taking into account the awareness, skill, knowledge, interest, and commitments of THM practitioners. Conversely, some respondents believed that the legal benchmarks were suitable and sufficient for regulating THM products, as long as they were effectively implemented. Hence, it is crucial for various stakeholders to collaborate in developing a comprehensive THM regulatory framework, regulatory tools, strategic plans, monitoring and evaluation mechanisms, research initiatives, training programs, and efforts to enhance the knowledge of THM healers, among other important matters. The effective implementation of THM product regulatory functions necessitates multidisciplinary collaboration, as recommended by the WHO (2018). It is concerning that only 7% of African countries possess a moderately developed capacity in this regard, while more than 90% have minimal or no capacity to fulfill their regulatory obligations. The study revealed challenges in the development and regulation of traditional herbal medicines (THMs), particularly in regional laboratories. The insufficiency of THM laboratories has a direct influence on TM research and development within a particular nation. The limited number of HM research laboratories is a widespread issue, as per the 2018 report by the WHO, which states that Africa has only 34 research institutions (WHO, 2018).

Various obstacles exist in the enforcement of regulations pertaining to herbal medicine. A study conducted by federal institution employees highlighted several challenges. These include the uncertainty of researchers and the scientific community toward traditional healers, as well as the limited interest and willingness of traditional medicine practitioners to adapt their practices and products. Furthermore, there is a shortage of trained personnel, financial resources, facilities, and quality control laboratories. The regulatory tools and instruments for traditional and herbal medicine are also deemed inadequate. Additionally, there was a lack of clear job descriptions, monitoring, and evaluation among stakeholders. Nationally, there is a dearth of well-structured organizational frameworks for regulating traditional medicine. The regulation of traditional medicine products prepared by healers in the first and second categories is undermined. Moreover, the EFDA occasionally exhibits delays in responding to applicants, particularly those applying for clinical trials. The implementation of the regulatory plan for traditional medicines is weak, as it remains mostly on paper without being put into action. Similar challenges were reported in studies from Kenya (Chebii et al., 2020) and Zimbabwe (Usai et al., 2021) and reports from Malaysia, India, and China (Goh, 2012).

In this study, the challenges were also evaluated at the regional health regulatory level. Pitfall challenges exist in the regional health regulatory environment: some traditional herbal medicine practitioners are not interested in registration, mix their THM practice with spiritual healing services, hide their knowledge, and do not print all the information needed on the product label; moreover, there is a lack of human resources, finance, research institutions, and quality control laboratories. Furthermore, there is an insufficiency of various THM regulatory tools, such as existing THM checklists, inadequate training, and a lack of support provided to healers and RHR workers. Therefore, it is imperative to implement an awareness-building initiative for traditional herbal remedies, along with establishing effective connections between governmental authorities and local personnel. When comparing the regional regulatory framework, it is less mature compared to the frameworks of Ghana, Uganda, Tanzania, Nigeria, and Mali (Kasilo et al., 2019), which are different from Ethiopia as they ratified the national THM practices and practitioners act and established an independent TMP council at the national level for THM practice and practitioners’ regulation.

In relation to the monitoring mechanisms for the quality, safety, and effectiveness of herbal medicine, the research discovered that most practitioners acknowledged the lack of standardized procedures, such as monographs and methods for gathering, preparing, and distributing their herbal medicine products. The participants in the study sourced medicinal plants from various locations, including their own gardens, rural areas, the local market, and even overseas. This was particularly evident in a sub-Saharan African nation where traditional herbal medicine services were solely provided through orally transmitted traditional medicine knowledge, as there was a dearth of official national pharmacopeias (Picking and Vandebroek, 2019). Hence, in order to uphold the standard of healthcare provisions, as it can lead to substantial disparities in the accessibility of HMs across nations with and without documented traditional medicine, herbal medicine, and HM practices, it is vital to establish pharmacopeias for THM and MPs.

All the study participants provided accounts of the harvested THM being transported by skilled individuals with great care. The HMs typically have a shelf life of 1 month to 1 year, depending on their nature and storage conditions. The expiration of THM products is determined through visual inspection, smell or flavor, changes in appearance, conducting tests, and considering other physical aspects of the HM. However, the label did not include specific product information, particularly the product name. This issue calls for urgent collaborative work to provide training and perform further research. The investigation revealed the presence of THM products presently accessible in the market; however, none of the herbal medicine practitioners were officially registered with the EFDA or validated by an authorized laboratory to ensure their intended quality, safety, and efficacy. Proof of evidence of the safety, efficacy, and quality of herbal medicines before they are granted MA is important to ensure the safety of consumers in the same way as for conventional medicines (Mssusa et al., 2023).

Moreover, the study additionally revealed that unregistered herbalists predominantly distribute THM, while registered herbalists continue to engage in activities that are legally prohibited. Furthermore, the majority of guidelines, formats, and checklists required for the regulation of TM products are still in the draft stage. These findings also highlight the significant delay in the practical regulation of TM products, practices, and practitioners in Ethiopia compared to countries such as Uganda, Ghana, India, and China (Cordell, 2012). Additionally, it indicates that the regulation of THMs (products, practices, and practitioners) in Ethiopia is currently not fully enforced; however, there are promising efforts underway to implement the herbal medicine regulatory framework.

## 6 Limitations and strengths of the study

The study had limitations in terms of its representation of the regulatory framework and its ability to provide scientific justification. This is because the study did not reflect the legal status of THM regulation or the regulatory practices of each regional state in the country. Furthermore, the study did not include lower-level regulatory offices, such as those found in the Oromia and SNNPR regional states, as well as manufacturers’ offices and other relevant offices. Additionally, the study only involved registered THM practitioners who practiced in the Oromia region and Addis Ababa city, excluding unregistered practitioners who did not participate in the study. The study does not take into account the viewpoints of consumers of traditional herbal medicines from consumer or patient advocacy organizations. These groups could be examined in a subsequent study.

The study’s key advantage lies in its capacity to assess the components of the regulatory framework for traditional herbal medicine products, including policy, proclamation, regulation, administrative structure, implementation of regulatory functions, challenges, and collaboration status in the regulation of herbal medicinal products, practices, and practitioners (healers).

## 7 Conclusion and recommendation

This study attempted to provide unwavering effort to the regulatory framework of traditional herbal medicine by providing valuable insights obtained from a comprehensive examination of policies, proclamations, regulations, administrative structures, implementation processes, registration procedures, and licensing requirements for traditional herbal medicinal products. These findings establish a solid legal foundation for herbal medicines and pave the way for the development of advanced systems that promote evidence-based practices and effective regulation of herbal medicines in Ethiopia. The existing national medicine proclamation regarding the regulation of THMs was found to be lacking and inadequate. Moreover, the findings revealed that regulatory agencies lack independent budget allocation, human resources, and facilities for effectively regulating traditional herbal medicine. Furthermore, despite efforts to enforce the regulatory framework, traditional herbal medicines (including products, practices, and practitioners) were not fully enforced in Ethiopia.

Hence, a collaboration of various stakeholders, including the Food and Drug Authority of Ethiopia, regional health regulatory offices, the Minister of Health, the Ethiopian Public Health Institution, and higher academic institutions, is required to join forces in order to establish a concrete regulatory framework for herbal medicine. This framework should not only focus on regulating traditional herbal medicine but also on fostering the growth and advancement of THM practices and products.

## Data Availability

The original contributions presented in the study are included in the article/Supplementary Material; further inquiries can be directed to the corresponding author.

## Abbreviations

THM, traditional herbal medicine; RHB, regional health bureau; RHR, regional health regulatory; SNNPR, the Southern Nations, Nationalities, and Peoples’ region; THMPs, traditional herbal medicine practitioners; MHROs, medicine and health regulatory offices; MoH, Ministry of Health; EFDA, Ethiopia Food and Drug Administration.
